# Multimorbidity and its association with mental health in an adult population of Peru

**DOI:** 10.17843/rpmesp.2024.414.13610

**Published:** 2024-10-21

**Authors:** Alejandro M. Angulo-Ramírez, Flavio C. Costa-Berlanga, Antonio Bernabé-Ortiz

**Affiliations:** 1 Universidad Científica del Sur, Lima, Peru. Universidad Científica del Sur Universidad Científica del Sur Lima Peru

**Keywords:** Chronic conditions, multimorbidity, depression, anxiety, stress, mental health

## Abstract

**Objective.:**

To evaluate the association between multimorbidity and mental health in adults aged 30 to 69 years.

**Materials and methods.:**

Secondary data analysis of a population-based study conducted in the peri-urban area of Tumbes in the northern coast of Peru. The dependent variables were: depressive symptoms, using the PHQ-9; anxiety symptoms, using the Goldberg scale; and perceived stress, assessed with the Cohen’s 14-question scale. The exposure variable was the presence of multimorbidity (presence or not of two or more chronic conditions and the number of chronic conditions). We assessed associations using crude and adjusted Poisson regression models.

**Results.:**

Data from 1600 participants were analyzed, mean age was 48.1 years (SD: 10.5), and 50.4% were women. The prevalence of multimorbidity was 15.9%, 23.3% presented depressive symptoms, 42.0% anxiety symptoms and 31.4% had high levels of perceived stress. The multivariable model showed that multimorbidity was associated with a higher prevalence of depressive symptoms (61%, 95%CI: 32% - 98%), anxiety symptoms (46%, 95%CI: 28% - 66%) and high (22%, 95%CI: 14% - 33%) but not moderate levels of perceived stress (6%; 95%CI: 0% - 12%). A higher number of chronic conditions was associated with higher prevalence of depressive symptoms, anxiety symptoms, and perceived stress levels.

**Conclusions.:**

The presence of multimorbidity is associated with a higher prevalence of depressive symptoms, anxiety symptoms, and perceived stress levels. Our results suggest the need for adequate mental health management in patients with multimorbidity.

## INTRODUCTION

Globally, mental health has taken great relevance as cases of depression, anxiety and stress have increased in recent years. Thus, the global standardized prevalence of anxiety disorders was 3780 per 100,000 individuals in 2019, while this number was 3440 per 100,000 individuals when it comes to depressive disorders ^1^. A systematic review that included 22 studies conducted during the pandemic (March 2020 to March 2021) reported that the prevalence of anxiety was 36.4%, that of depression was 26.6%, and that of stress was 42% ^2^. Peru is no stranger to this situation. For example, a study conducted on 1699 participants during the COVID-19 pandemic reported that 59.7% presented symptoms of psychological distress ^3^.

On the other hand, multimorbidity, defined as the coexistence of two or more chronic diseases in the same individual, appears to be more frequent in low- and middle-income countries ^4^, whereas in high-income countries it appears at older ages. Thus, a cross-sectional study using data from medical services in Scotland showed that the prevalence of multimorbidity was 23% using a list of 40 conditions, and was more prevalent in older adults ^5^. However, the prevalence of multimorbidity in Latin America and the Caribbean can be as high as 43% ^6^, meanwhile the prevalence of this condition in Peru has been estimated at 19% ^7^, ranging from 14% to 23% depending on the region evaluated. The prevalence of multimorbidity is usually heterogeneous due to the diverse number of chronic conditions that are used to define it, as well as the epidemiological profile of each country ^8^.

In the general population, the presence of more than one chronic disease at the same time is associated with several factors, including anxiety disorders ^9^, and thus deterioration of mental health. On the other hand, a systematic review reported that subjects with multimorbidity have up to twice the risk of suffering from depressive disorder compared to people without multimorbidity ^10^. The heterogeneity of multimorbidity in the region ^6^, and the association of physical multimorbidity and mental health deterioration in other countries of the region ^11^ makes it imperative to evaluate the association between multimorbidity and mental health, especially in areas of scarce economic resources such as peri-urban areas, since many chronic conditions are not detected in time. Depression and anxiety are usually included as part of the definition of multimorbidity and the combination of physical and mental conditions can result in an overload of the health system, including an increase in the demand for mental health care and human resources. Moreover, the health system in Peru usually treats conditions individually rather than in an integrated manner.

Therefore, the aim of this study was to evaluate the association between the presence of multimorbidity and certain mental health indicators (depressive symptoms, anxiety and perceived stress) in adult patients aged 30 to 69 years in a region in northern Peru.

**KEY MESSAGES**

Motivation for the study. The presence of multimorbidity is increasingly frequent in the general population, and this may be associated with mental health problems.

Main findings. The presence of multimorbidity, and a great number of chronic conditions, was associated with a higher prevalence of depressive symptoms, anxiety and perceived stress in an adult population in a region of northern Peru.

Implications. Our findings suggest the need for adequate mental health management in patients with more than one chronic disease.

## MATERIALS AND METHODS

### Study design

A secondary data analysis of a population-based, cross-sectional, analytical study was performed to evaluate two diagnostic methods for detecting cases of diabetes at the population level ^12^.

### Population and study area

The study was conducted in the peri-urban area of Tumbes located on the northern coast of Peru. Tumbes has approximately 225 thousand inhabitants according to the 2017 national census, 90% live in urban area ^13^, and about 20% and 1% of its population are considered poor and extremely poor, respectively, and 15% do not have basic sanitation services.

The study population included adults between 30 and 69 years of age, of both sexes, habitual and full-time residents of the study area (≥6 months), and capable of understanding the procedures and giving informed consent. Women who reported being pregnant, persons with any disability for taking anthropometric measurements, and those bedridden were excluded from the study. For this analysis, we used all the data from the original study (n = 1609) and excluded those records that did not have complete information on the variables of interest (multimorbidity and mental health).

The original study sample was drawn randomly, stratified by sex, using the most current census information for the study area for participant selection (2014 regional census). Only one participant per household was selected in order to avoid clustering of risk factors.

### Definition of variables

The independent variable was multimorbidity, defined as the coexistence of two or more chronic diseases in the same person ^8^, chosen from a list of ten conditions that were evaluated objectively or subjectively in the original study. The five conditions assessed objectively were: arterial hypertension (systolic pressure ≥140 mmHg or diastolic pressure ≥90 mmHg or with previous medical diagnosis) ^14^; type 2 diabetes mellitus (fasting glucose ≥126 mg/dL or postprandial glucose ≥200 mg/dL after 2 hours post ingestion of anhydrous glucose, or previous medical diagnosis) ^15^; stroke, defined by a validated questionnaire (at least three positive responses) ^16^; periodontitis, defined by a validated questionnaire (more than four items) ^17^; and Parkinson's disease, defined by the Duarte screening scale (≥ 42 items) ^18^. The other five conditions on the list were self-reported based on a previous medical diagnosis: arrhythmia, heart failure, cancer, dyslipidemia, and acute myocardial infarction. In addition to the presence of multimorbidity, the number of chronic conditions each participant had was also assessed (0 [none], 1, 2, and ≥ 3).

Three mental health variables were evaluated as dependent variables. The first was the presence of depressive symptoms, assessed with the PHQ-9 scale, an instrument that has a total of nine questions with four response options each with a score of 0 to 3 points (from never to almost every day). The scale has been validated in Spanish in Chile ^19^^)^ and Peru ^20^, and uses a cut-off point ≥4 points to define the presence of depressive symptoms ^21^. The second variable was the presence of anxiety symptoms, evaluated through the Goldberg scale, which presents 9 questions with two response options (yes and no) and those with ≥ 4 points are considered as positive ^22^. Finally, the third variable was perceived stress, assessed through the Cohen Perceived Stress Scale, a 14-question scale with five response options (never, rarely, sometimes, many times and always). The score per question ranges from 0 to 4, with a minimum score of 14 and a maximum of 70 points ^23^. The total score was divided into tertiles (high, medium and low) for analysis and the lowest category was used as reference.

The covariates included in the analysis were sociodemographic, such as: age (30-39, 40-49, 50-59 and 60 or older); sex (male and female); level of primary education (less than 7 years), secondary education (between 7 and 11 years) and higher education (12 years and older); and socioeconomic status, based on a composite measure of each household’s standard of living that is calculated with data collected on the respondent’s household goods and services (e.g., television, bicycle, television, roof, wall and floor material, etc.). This procedure is based on DHS Program techniques that are almost common to all countries participating in the DHS Program ^24^. All these indicators were weighted, constructing a numerical wealth index categorized into terciles (low, medium and high) for this analysis. Other covariates were also included, such as: current job (yes or no); having health insurance (yes or no); habitual smoker, defined as smoking at least one cigarette daily (yes or no); alcohol abuse, using the Alcohol Use Disorder Identification Test (AUDIT: dependent ≥8 or nondependent <8) ^25^; and physical activity levels, based on the short version of the International Physical Activity Questionnaire or IPAQ (low or moderate/high).

### Procedures

With prior consent, the study questionnaire was applied. This was based on the World Health Organization (WHO) STEPS instrument (describes the collection, analysis and dissemination of information about risk factors for noncommunicable diseases in individuals aged 18 to 64 years). The questionnaire was applied using the Open Data Kit (ODK) software using tablets to obtain demographic, behavioral, personal and family medical history data oriented to glucose metabolic alterations. For blood pressure measurement, an automated monitor was used after a five-minute rest period with the feet on the floor and the back resting on a chair ^14^. Three blood pressure measurements were taken at least one minute apart. The average of the second and third measurements were used for blood pressure estimation. On the other hand, previously trained laboratory personnel were in charge of performing the oral glucose tolerance test for each participant; the basal and postprandial glucose values were used to estimate the prevalence of type 2 diabetes mellitus.

### Statistical analysis

The data were analyzed using the statistical program Stata 16 for Windows. First, a description of the study population was made using mean and standard deviation (SD) for numerical variables, on the other hand absolute and relative frequencies were used for categorical variables. Then, prevalence and 95% confidence intervals (95%CI) were estimated for the variables of interest (multimorbidity, depressive symptoms, anxiety symptoms, and perceived stress). Comparisons between categorical variables were made with the Chi-square test. Finally, to determine the association of our variables, we created Poisson Regression models with robust variance, both crude and adjusted, using epidemiological criteria based on similar previous study references ^26^^-^^28^, and prevalence ratios (PR); 95%CI were reported. Poisson regression was also used for perceived stress, comparing those in the middle tercile and upper tercile against the lower tercile (used as a reference group).

### Ethical Aspects

The Universidad Peruana Cayetano Heredia, Peru, and the London School of Hygiene and Tropical Medicine, United Kingdom, reviewed and approved the original study protocol. This protocol was submitted to and approved by the Ethics Committee of the Universidad Científica del Sur (registration code: PRE-15-2023-00297).

## RESULTS

### Overview of the study population

A total of 2114 people were invited to participate, and 1609 records were part of the original study, but only data from 1600 (75.6%) were analyzed (9 records did not have the data of interest and were eliminated). The population consisted of 807 (50.4%) women and had a mean age of 48.1 years (SD: 10.5). Most participants (46.4%) had completed high school.

### Characteristics of the population according to the number of chronic diseases and the presence of multimorbidity

Only 255 participants (15.9%; 95%CI: 14.1% - 17.8%) had multimorbidity while 51% did not have any disease. Being female (p=0.001) and being older (p<0.001) were associated with a higher number of chronic conditions and multimorbidity. On the other hand, having a lower level of education (p<0.001), not having alcohol abuse (p=0.004) and low levels of physical activity (p=0.003) were associated with greater multimorbidity (Table 1).

**Table 1:** Characteristics of the study population according to the presence of multimorbidity and the number of chronic conditions.

		Multimorbidity			Number of chronic conditions				
		Total= 1345 (%)	Yes=255 (%)	p-b value	0=822 (%)	1=523 (%)	2=187 (%)	>3=68 (%)	p-value
Sex									
	Female	653 (48.6)	154 (60.4)	<0.001	382 (46.5)	271 (51.8)	109 (58.3)	45 (66.2)	<0.001
	Male	692 (51.4)	101 (39.6)		440 (53.5)	252 (48.2)	78 (41.7)	23 (33.8)	
Age									
	30-39 years	416 (30.9)	21 (8.2)	<0.001	315 (38.3)	101 (19.4)	17 (9.1)	4 (5.9)	<0.001
	40-49 years	434 (32.3)	45 (17.7)		253 (30.8)	181 (34.6)	37 (19.8)	8 (11.7)	
	50-59 years	308 (22.9)	99 (38.8)		165 (20.1)	143 (27.3)	70 (37.4)	29 (42.7)	
	60+ years	187 (13.9)	90 (35.3)		89 (10.8)	98 (18.7)	63 (33.7)	27 (39.7)	
Education level									
	Primary school	394 (29.3)	123 (48.2)	<0.001	210 (25.6)	184 (35.2)	79 (42.3)	44 (64.7)	<0.001
	Secondary school	642 (47.7)	101 (39.6)		414 (50.4)	228 (43.6)	83 (44.4)	18 (26.5)	
	Higher education	309 (23.0)	31 (12.2)		198 (24.0)	111 (21.2)	25 (13.4)	6 (8.8)	
Socioeconomic level									
	Low	446 (33.2)	92 (36.1)	0.650	267 (32.5)	179 (34.2)	65 (34.8)	27 (39.7)	0.220
	Middle	461 (34.3)	85 (33.3)		303 (36.8)	158 (30.2)	63 (33.7)	22 (32.4)	
	High	438 (32.5)	78 (30.6)		252 (30.7)	186 (35.6)	59 (31.5)	19 (27.9)	
Currently employed									
	Yes	937 (69.7)	149 (58.4)	<0.001	577 (70.2)	360 (68.8)	121 (64.7)	28 (41.2)	<0.001
Health insurance									
	Yes	1224 (91.0)	238 (93.3)	0.220	746 (90.7)	478 (91.4)	170 (90.9)	68 (100.0)	0.080
Daily smoking									
	Yes	81 (6.0)	11 (4.3)	0.280	54 (6.6)	27 (5.2)	8 (4.3)	3 (4.4)	0.510
Alcohol abuse									
	Yes	111 (8.3)	8 (3.1)	0.004	78 (9.5)	33 (6.3)	6 (3.2)	2 (2.9)	0.005
Physical activity									
	Moderate/high	860 (63.9)	138 (54,1)	0.003	534 (65.0)	326 (62.3)	107 (57.2)	31 (45.6)	0.006
	Low	485 (36.1)	117 (45.9)		288 (35.0)	197 (37.7)	80 (42.8)	37 (54.4)	

### Characteristics of the population according to mental health indicators

The prevalence of depressive symptoms was 23.3% (95%CI: 21.1% - 25.3%) and was more frequent in women (p<0.001), in those with a greater number of chronic conditions (p<0.001) and in those with multimorbidity (p<0.001). On the other hand, the prevalence of anxiety symptoms was 42.0% (95%CI: 39.5% - 44.4%) and was associated with female sex (p<0.001), no smoking (p=0.011), no alcohol abuse (p=0.004), a greater number of chronic conditions (p<0.001) and the presence of multimorbidity (p<0.001). Finally, perceived stress was more frequent in women (p<0.001), in those who did not smoke daily (p=0.003), in people who had a greater number of chronic conditions (p<0.001) and in those with multimorbidity (p<0.001). See details in Table 2.

**Table 2:** Characteristics of the study population according to mental health indicators (depressive, anxiety and stress symptoms).

			Depressive symptoms		Anxiety symptoms		Perceived stress	
		n	Yes=327 (%)	p-value	Yes=672 (%)	p-value	High=503 (%)	p-value
Sex								
	Female	807	276 (34.2)	<0.001	452 (56.0)	<0.001	339 (42.0)	<0.001
	Male	793	96 (12.1)		220 (27.7)		164 (20.6)	
Age								
	30-39 years	437	95 (21.7)	0.16	173 (39.5)	0.03	130 (29.7)	0.47
	40-49 years	479	108 (22.5)		205 (42.8)		162 (33.8)	
	50-59 years	407	111 (27.2)		192 (47.1)		130 (31.9)	
	60+ years	277	58 (20.9)		102 (36.8)		81 (29.2)	
Education level								
	Primary school	517	136 (26.3)	0.14	223 (43.1)	0.59	182 (35.2)	0.002
	Secondary school	743	162 (21.8)		302 (40.7)		239 (32.2)	
	Higher education	340	74 (21.8)		147 (43.2)		82 (24.1)	
Socioeconomic level								
	Low	538	132 (24.5)	0.49	232 (43.1)	0.68	190 (35.3)	0.05
	Middle	546	129 (23.6)		231 (42.3)		164(30.0)	
	High	516	111 (21.5)		209 (40.5)		149 (28.9)	
Currently employed								
	No	514	179 (34.8)	<0.001	275 (53.5)	<0.001	216 (42.0)	<0.001
	Yes	1086	193 (17.8)		397 (36.6)		287 (26.4)	
Health insurance								
	No	138	27 (19.6)	0.28	49 (35.5)	0.11	30 (21.7)	0.01
	Yes	1462	345 (23.6)		623 (42.6)		473 (32.4)	
Daily smoking								
	No	1508	357 (23.7)	0.10	645 (42.8)	0.01	487 (32.3)	0.003
	Yes	92	15 (16.3)		27 (29.4)		16 (17.4)	
Alcohol abuse								
	No	1481	351 (23.7)	0.13	637 (43.0)	0.004	475 (32.1)	0.05
	Yes	118	21 (17.7)		35 (29.4)		28 (23.5)	
Physical activity								
	Moderate/high	998	199 (19.9)	<0.001	389 (39.0)	0.002	273 (27.4)	<0.001
	Low	602	173 (28.7)		283 (47.0)		230 (38.2)	
Number of chronic conditions								
	0	822	166 (20.2)	<0.001	290 (35.3)	<0.001	217 (26.4)	<0.001
	1	523	112 (21.4)		231 (44.2)		167 (31.9)	
	2	187	59 (31.6)		102 (54.6)		76 (40.6)	
	3+	68	35 (51.5)		49 (72.1)		43 (63.2)	
Multimorbidity								
	No	1345	278 (20.7)	<0.001	521 (38.7)	<0.001	384 (28.6)	<0.001
	Yes	255	94 (36.9)		151 (59.2)		119 (46.7)	

### Association between multimorbidity and mental health

The multivariable model showed that the presence of multimorbidity was associated with a higher prevalence of depressive symptoms (61%; 95%CI: 32% - 98%) and a higher prevalence of anxiety symptoms (46%; 95%CI: 28% - 66%). While the presence of multimorbidity was associated with high levels of stress (22%; 95%CI: 14% - 33%), it was not associated with moderate levels (6%; 95%CI: 0% - 12%). On the other hand, a greater number of chronic conditions was associated with a higher prevalence of depressive symptoms, anxiety symptoms and perceived stress (Table 3).

**Table 3:** Association between multimorbidity, number of chronic conditions and mental health: crude and adjusted models.

		Multimorbidity	Number of chronic conditions		
		Yes vs. No (95%CI)	1 vs. 0 (95%CI)	2 vs. 0 (95%CI)	>3 vs. 0 (95%CI)
Depressive symptoms					
	Crude model	1.78 (1.47 - 2.16)	1.06 (0.86 - 1.31)	1.56 (1.22 - 2.01)	2.55 (1.95 - 3.33)
	Adjusted model*	1.61 (1.32 - 1.98)	1.03 (0.83 - 1.27)	1.44 (1.11 - 1.87)	2.14 (1.60 - 2.89)
Anxiety symptoms					
	Crude model	1.53 (1.35 - 1.73)	1.25 (1.10 - 1.43)	1.54 (1.31 - 1.81)	2.04 (1.71 - 2.43)
	Adjusted model*	1.46 (1.28 - 1.66)	1.23 (1.08 - 1.40)	1.50 (1.27 - 1.77)	1.93 (1.61 - 2.32)
Perceived stress (moderate vs. low)					
	Crude model	1.07 (1.01 - 1.12)	0.99 (0.96 - 1.04)	1.03 (0.96 - 1.10)	1.23 (1.13 - 1.33)
	Adjusted model*	1.05 (0.99 - 1.12)	0.99 (0.95 - 1.04)	1.02 (0.95 - 1.09)	1.20 (1.11 - 1.30)
Perceived stress (high vs. low)					
	Crude model	1.26 (1.17 - 1.34)	1.07 (0.99 - 1.15)	1.20 (1.09 - 1.31)	1.54 (1.44 - 1.66)
	Adjusted model*	1.22 (1.14 - 1.30)	1.05 (0.98 - 1.12)	1.16 (1.07 - 1.27)	1.49 (1.37 - 1.63)

The models evaluate each outcome (in the right-hand column) according to each separate exposure (in the columns).

* Model adjusted for sex, age, education level, socioeconomic status, currently employed, health insurance, daily smoking, alcohol abuse and daily physical activity.

## DISCUSSION

Our findings show that both multimorbidity and a greater number of chronic conditions were associated with a higher prevalence of depressive symptoms, anxiety and perceived stress. On the other hand, 15% of the studied population presented multimorbidity, while a large part of the population had symptoms of depression, anxiety and perceived stress.

Several studies show that multimorbidity is associated with several mental health problems. For example, according to the results of a relatively recent systematic review ^26^, both anxiety and depression were more frequent in people with multimorbidity. Regarding anxiety, two previous studies corroborate our findings. Thus, a U.S. study including 4219 older adults reported that a greater number of chronic conditions are associated with an increased risk of anxiety ^27^. Similarly, another study recruiting 1315 participants with obesity in Canada reported that an increased number of chronic conditions was associated with the presence of mental health symptoms ^11^.

Regarding depression, one study showed that the risk of depressive disorder was twice as high for people with multimorbidity compared to those without multimorbidity and three times higher for people with multimorbidity compared to those without any chronic physical condition ^10^. This finding is corroborated by a study from South Africa with a sample of 2549 participants, which determined that there is a higher prevalence of depressive symptoms in those with multimorbidity ^29^.

Finally, regarding perceived stress, two studies were also in line with our findings. A study of 34,129 participants in six countries, concluded that multimorbidity is associated with higher levels of stress in low- and middle-income older adults ^30^. The other study involving 229,293 adults living in communities in 44 low- and middle-income countries found that multimorbidity was associated with a higher prevalence of perceived stress ^31^.

It is considered that multimorbidity is a challenge in the field of public health, and that the affectation of mental health due to the increase in the number of chronic conditions that is occurring may have a relevant impact. This can be important in countries with limited resources such as Peru due to the lack of a comprehensive approach to the conditions presented by patients. In this case, although management should be cross-cutting, specialists usually evaluate only the conditions that are relevant to them without the need to verify their impact on other aspects of the patient’s life. In this context, an individual should be considered as a biopsychosocial being and therefore appropriately manage both physical and mental health.

Our results, such as those from other studies, suggest that an appropriate system for a multidisciplinary approach for patients with multimorbidity should be implemented. We believe that understanding the association between the presence of multiple chronic conditions and mental health is essential to provide more comprehensive health care, improve treatment outcomes and quality of life for patients, as well as to inform health policies and strategies effectively.

The COVID-19 pandemic exposed all the deficiencies of our health system that affect the design of care strategies, prevention and, evaluation of health policies and research, and epidemiological studies related to multimorbidity ^32^. In addition, the pandemic highlighted the need to invest resources in mental health in Peru ^33^, therefore, it is essential that both the authorities and society as a whole recognize the need to address mental health challenges in a comprehensive and effective manner.

An important strength of our study lies in its population representativeness and in the objective assessment of several of the included chronic conditions, especially those most prevalent. However, there are some limitations that deserve discussion. First since this was a cross-sectional study, only association and not causality could be assessed. Additionally, reverse causality could be present, that is, the presence of mental health symptoms could increase the probability of having multimorbidity. Then, the list of diseases used for the definition of multimorbidity may be very different in other regions, which would affect the results, however, it was grouped using the definition of multimorbidity that is widely recognized, in addition to being assessed according to the number of chronic conditions. Moreover, some chronic conditions were self-reported which could have an impact on the estimates. However, many of them were the least common at the population level (arrhythmia, heart failure, cancer and acute myocardial infarction). Finally, there are more mental health conditions that may be related to multimorbidity such as psychosis, post-traumatic stress disorder (PTSD) and substance abuse disorders, which were not assessed.

In conclusion, our results show that the presence of multimorbidity was associated with a higher prevalence of depressive symptoms, anxiety and perceived stress. On the other hand, a greater number of chronic conditions was also associated with a higher presence of these symptoms. These results suggest the need for adequate mental health management in patients with multimorbidity.
