# Supplementary material for: Multimorbidity and its association with mental health in an adult population of Peru
Source: Rev Peru Med Exp Salud Publica. 2024 Oct 21;41(4):343–50. doi: 10.17843/rpmesp.2024.414.13610 (PMC11797581; doi:10.17843/rpmesp.2024.414.13610)
Supplement: Translation

# Translation: Multimorbilidad y su asociación con la salud mental en una población adulta del Perú

**Authors:** Alejandro M. Angulo-Ramírez, Flavio C. Costa-Berlanga, Antonio Bernabé-Ortiz

**Affiliations:** 1 Universidad Científica del Sur, Lima, Perú.

**Keywords:** Enfermedades no transmisibles, multimorbilidad, depresión, ansiedad, estrés, salud mental

## Abstract

**Objetivos.:**

Evaluar la asociación entre multimorbilidad y salud mental en personas adultas de 30 a 69 años.

**Materiales y métodos.:**

Análisis de datos secundarios de un estudio poblacional realizado en la zona periurbana de Tumbes en la costa norte del Perú. Las variables dependientes fueron: síntomas depresivos, usando el PHQ-9; síntomas de ansiedad, usando la escala de Goldberg; y estrés percibido, evaluado usando la escala de 14 preguntas de Cohen; mientras que la exposición fue la presencia de multimorbilidad (presencia o no de dos o más condiciones crónicas y el número de condiciones crónicas). Las asociaciones se evaluaron usando modelos regresión de Poisson crudos y ajustados.

**Resultados.:**

Se analizaron datos de 1600 participantes, edad media de 48,1 años (DE: 10,5), 50,4% fueron mujeres. La prevalencia de multimorbilidad fue de 15,9%, el 23,3% presentaron síntomas depresivos, 42,0% síntomas de ansiedad y 31,4% tenían niveles altos de estrés percibido. En modelo multivariable, la presencia de multimorbilidad estuvo asociada a una mayor prevalencia de síntomas depresivos (61%, IC95%: 32% - 98%), síntomas de ansiedad (46%, IC95%: 28% - 66%) y niveles altos (22%, IC95%: 14% - 33%), pero no moderados de estrés percibido (6%; IC95%: 0% - 12%). Un mayor número de condiciones crónicas se asoció a mayor prevalencia de síntomas depresivos, síntomas de ansiedad y niveles de estrés percibido.

**Conclusiones.:**

La presencia de multimorbilidad se asocia a una mayor prevalencia de síntomas depresivos, de ansiedad y niveles de estrés percibido. Nuestros resultados sugieren la necesidad de un manejo adecuado de la salud mental en pacientes con multimorbilidad.

## INTRODUCCIÓN

A nivel mundial, la salud mental ha tomado gran relevancia dado que los casos de depresión, ansiedad y estrés se han elevado en los últimos años. Así, la prevalencia global estandarizada de desórdenes de ansiedad fue de 3780 por cada 100,000 individuos en el 2019, mientras que este número fue de 3440 por 100,000 individuos en el caso de desórdenes depresivos ^1^. En una revisión sistemática que incluyó 22 estudios realizados durante la pandemia (marzo 2020 a marzo 2021) reportó que la prevalencia de ansiedad fue de 36,4%, la de depresión fue de 26,6%, y la de estrés de 42% ^2^. El Perú no se encuentra ajeno a esta situación. Por ejemplo, un estudio realizado en 1699 participantes, durante la pandemia del COVID-19, reportó que el 59,7% presentaron síntomas de distrés psicológico ^3^.

De otro lado, la multimorbilidad, definida como la coexistencia de dos o más enfermedades crónicas en un mismo individuo, parece ser más frecuente en los países de ingresos bajos y medios ^4^, mientras que, en países de altos ingresos, aparece a mayor edad. Así, un estudio transversal utilizando los datos de los servicios médicos en Escocia mostró que la prevalencia de multimorbilidad fue del 23% usando una lista de 40 condiciones, y era más prevalente en adultos mayores ^5^. Sin embargo, la prevalencia de multimorbilidad en Latino América y el Caribe puede llegar hasta el 43% ^6^, mientras que la prevalencia de esta condición en Perú se ha estimado en 19% ^7^, oscilando entre 14% y 23% dependiendo de la región evaluada. La prevalencia de multimorbilidad suele ser heterogénea debido al diverso número de condiciones crónicas evaluadas que se utilizan para definirla, así como el perfil epidemiológico de cada país ^8^.

En la población general, la presencia de más de una enfermedad crónica a la vez está asociada con diversos factores, incluyendo trastorno ansiosos ^9^, y por ende deterioro de la salud mental. De otro lado, una revisión sistemática reportó que los sujetos con multimorbilidad tienen hasta el doble de riesgo de padecer trastorno depresivo comparado con personas sin multimorbilidad ^10^. La heterogeneidad de la multimorbilidad en la región ^6^, y la asociación de multimorbilidad física y deterioro de salud mental en otros países de la región ^11^, hace imperativo evaluar la asociación entre multimorbilidad y la salud mental, especialmente en zonas de escasos recursos económicos como áreas periurbanas, ya que muchas de las condiciones crónicas no son detectadas a tiempo. Usualmente depresión y ansiedad son incluidas como parte de la definición de multimorbilidad y la unión de condiciones físicas como mentales puede terminar en una sobrecarga del sistema de salud, incluyendo un incremento en la demanda de atenciones y recursos humanos en salud mental. Más aún, usualmente el sistema de salud en Perú atiende y trata condiciones en forma individual en vez de hacerlo en forma integrada.

Por lo tanto, el objetivo del presente estudio fue evaluar la asociación entre la presencia de multimorbilidad y ciertos indicadores de salud mental (síntomas depresivos, de ansiedad y de estrés percibido) en pacientes adultos de 30 a 69 años en una región en el norte del Perú.

**MENSAJES CLAVE**

Motivación para realizar el estudio. En población general, la presencia de multimorbilidad es cada vez más frecuente, y esto puede estar asociado a problemas de salud mental.

Principales hallazgos. La presencia de multimorbilidad, y un mayor número de condiciones crónicas, se asoció a una mayor prevalencia de síntomas depresivos, de ansiedad y de estrés percibido en una población adulta de una región del norte del Perú.

Implicancias. Nuestros hallazgos sugieren la necesidad de un manejo adecuado de la salud mental en pacientes con más de una enfermedad crónica.

## MATERIALES Y MÉTODOS

### Diseño de estudio

Se realizó un análisis de datos secundario de un estudio poblacional, transversal y analítico que tuvo como objetivo evaluar dos métodos diagnósticos para detectar casos de diabetes a nivel poblacional ^12^.

### Población y lugar de estudio

El estudio se realizó en la zona periurbana de Tumbes ubicada en la costa norte del Perú. Tumbes tiene aproximadamente 225 mil habitantes según el censo nacional del 2017, el 90% viven en zona urbana ^13^, y cerca del 20% y 1% de su población es considerada pobre y extremadamente pobre, respectivamente, y el 15% no tienen los servicios sanitarios básicos.

La población de estudio incluyó personas adultas entre 30 y 69 años, de ambos sexos, residentes habituales y a tiempo completo de la zona de estudio (≥6 meses), y capaces de comprender los procedimientos y dar su consentimiento informado. Las mujeres que reportaron estar en estado de gestación, las personas con alguna discapacidad para la toma de medidas antropométricas y aquellas postradas en cama fueron excluidas del estudio. Para el presente análisis se utilizó la totalidad de datos provenientes del estudio original (n = 1609) y se excluyeron aquellos registros que no tuvieron información completa de las variables de interés (multimorbilidad y salud mental).

La muestra del estudio original fue tomada en forma aleatoria, estratificada por sexo, usando la información del censo más actual del área de estudio para la selección de los participantes (censo regional del año 2014). Con la finalidad de evitar el aglomeramiento de factores de riesgo, sólo se seleccionó un participante por vivienda.

### Definición de variables

La variable independiente fue multimorbilidad, definida como la coexistencia de dos o más enfermedades crónicas en una misma persona ^8^, escogidas de una lista potencial de diez condiciones que fueron evaluadas objetiva o subjetivamente en el estudio original. Las cinco condiciones evaluadas en forma objetiva fueron: hipertensión arterial (presión sistólica ≥140 mmHg o diastólica ≥90 mmHg o con diagnóstico médico previo) ^14^; diabetes mellitus tipo 2 (glucosa en ayunas ≥126 mg/dL o glucosa postprandial ≥200 mg/dL después de 2 horas post ingesta de glucosa anhidra, o diagnóstico médico previo) ^15^; accidente cerebrovascular, definido por un cuestionario validado (al menos tres respuestas positivas) ^16^; periodontitis, definida por un cuestionario validado (más de cuatro puntos) ^17^, y enfermedad de Parkinson, definida por la escala de tamizaje de Duarte (≥ 42 puntos) ^18^. Las otras cinco condiciones de la lista fueron auto reportadas en base a un diagnóstico médico previo: arritmia, insuficiencia cardiaca, cáncer, dislipidemia e infarto agudo de miocardio. Además de la presencia de multimorbilidad, también se evaluó el número de condiciones crónicas que tenía cada participante (ninguna [0], 1, 2 y ≥ 3).

Tres fueron las variables de salud mental evaluadas como variables dependientes. La primera fue la presencia de síntomas depresivos, evaluados con la escala PHQ-9, instrumento que cuenta con un total de nueve preguntas con cuatro opciones de respuesta cada una con un puntaje de 0 a 3 puntos (desde nunca hasta casi todos los días). La escala ha sido validada al español en Chile ^19^ y en Perú ^20^, y usa un punto de corte ≥4 puntos para definir la presencia de síntomas depresivos ^21^. La segunda variable fue la presencia de síntomas de ansiedad, evaluados a través de la escala de Goldberg, que presenta 9 preguntas con dos opciones de respuesta (sí y no) y aquellos con ≥ 4 puntos se considera como positivo ^22^. Finalmente, la tercera variable fue estrés percibido, evaluado mediante la Escala de Estrés Percibido de Cohen, una escala de 14 preguntas con cinco opciones de respuesta (nunca, raras veces, algunas veces, muchas veces y siempre). El puntaje por pregunta va de 0 a 4, con un puntaje mínimo de 14 y máximo de 70 puntos ^23^. El puntaje total fue dividido en terciles (alto, medio y bajo) para el análisis y se usó la categoría más baja como referencia.

Las covariables incluidas en el análisis fueron sociodemográficas como: edad (de 30-39, 40-49, 50-59 y 60 o más años); sexo (masculino y femenino); nivel de educación primaria (menos de 7 años), secundaria (entre 7 y 11 años) y superior (12 años a más); y nivel socioeconómico, basado en una medida compuesta del nivel de vida de cada hogar que se calcula con datos recopilados sobre los bienes y servicios del hogar del encuestado (por ejemplo, televisión, bicicleta, televisión, material del techo, de las paredes y del piso, etc.). Este procedimiento se basa en técnicas del Programa DHS que son casi comunes a todos los países que participan en dicho programa ^24^. Todos estos indicadores fueron ponderados, construyendo un índice numérico de riqueza categorizado en terciles (bajo, medio y alto) para este análisis. También se incluyeron otras covariables como: trabajo actual (sí o no); tener seguro de salud (sí o no); fumador habitual, definido como el consumo de al menos un cigarrillo diario (sí o no); abuso de alcohol, utilizando la Prueba de Identificación para el Desorden de Consumo de Alcohol (AUDIT: dependiente ≥8 o no dependiente <8) ^25^; y niveles de actividad física, en base a la versión corta del Cuestionario Internacional de Actividad Física o IPAQ (bajo o moderado/alto).

### Procedimientos

Previo consentimiento, se aplicó el cuestionario de estudio. Este estuvo basado en el instrumento STEPS (posibilita la recopilación, análisis y divulgación de información acerca de los factores de riesgo de enfermedades no transmisibles en individuos de 18 a 64 años) de la Organización Mundial de la Salud (OMS). El cuestionario se aplicó usando el software Open Data Kit (ODK) mediante tabletas para obtener datos demográficos, conductuales, antecedentes médicos personales y familiares orientados a las alteraciones metabólicas de la glucosa. Para la medida de la presión arterial, se utilizó un monitor automatizado luego de un periodo de reposo de cinco minutos con los pies apoyados en el piso y la espalda recostada en una silla ^14^. Se realizaron tres tomas de la presión arterial separadas al menos un minuto entre ellas. El promedio de la segunda y la tercera medida fueron utilizados para la estimación de la presión arterial. Por otro lado, personal de laboratorio capacitado previamente se encargó de realizar la prueba de tolerancia oral a la glucosa en cada participante y los valores de glucosa basal y postprandial fueron usadas para estimar la prevalencia de diabetes mellitus tipo 2.

### Análisis estadístico

La data fue analizada utilizando el programa estadístico Stata 16 para Windows. En primer lugar, se realizó una descripción de la población del estudio usando la media y desviación estándar (DE) para las variables numéricas, mientras que se usó frecuencias absolutas y relativas para variables categóricas. Luego, se estimó la prevalencia e intervalos de confianza al 95% (IC95%) de las variables de interés (multimorbilidad, síntomas de depresión, síntomas de ansiedad y estrés percibido). Las comparaciones entre variables categóricas se hicieron con la prueba Chi cuadrado. Finalmente, para observar la asociación de nuestras variables se crearon modelos de Regresión de Poisson con varianza robusta, tanto crudos y ajustados, usando el criterio epidemiológico basado en referencias de estudio previos similares ^26^^-^^28^, y reportándose razones de prevalencia (RP) e IC95%. En el caso de estrés percibido también se usó regresión de Poisson, comparando aquellos en el tercil medio y el tercil superior contra el tercil bajo (usado como grupo de referencia).

### Aspectos éticos

La Universidad Peruana Cayetano Heredia, Perú, y London School of Hygiene and Tropical Medicine, Reino Unido, realizaron la revisión y aprobación del protocolo original del estudio. El presente protocolo fue presentado y aprobado por el Comité de Ética de la Universidad Científica del Sur (código de registro: PRE-15-2023-00297).

## RESULTADOS

### Descripción general de la población de estudio

Un total de 2114 personas fueron invitadas a participar, y 1609 registros formaron parte del estudio original, pero solo los datos de 1600 (75,6%) fueron analizados (9 registros no tenían los datos de interés y fueron eliminados). La población estuvo conformada por 807 (50,4%) mujeres y tuvo una media de edad de 48,1 años (DE 10,5). La mayoría de participantes (46,4%) tuvo secundaria completa.

### Características de la población según el número de enfermedades crónicas y la presencia de multimorbilidad

Sólo 255 participantes (15,9%; IC95%: 14,1% - 17,8%) presentaron multimorbilidad mientras que el 51% no presentó ninguna enfermedad. Ser mujer (p=0,001) y tener mayor edad (p<0,001) se asociaron a un mayor número de condiciones crónicas y multimorbilidad. De otro lado, tener menor nivel de educación (p<0,001), no presentar abuso de alcohol (p=0,004) y los bajos niveles de actividad física (p=0,003) se asociaron a una mayor multimorbilidad (Tabla 1).

**Tabla 1:** Características de la población de estudio según la presencia de multimorbilidad y el número de condiciones crónicas.

		Multimorbilidad			Número de condiciones crónicas				
		Total= 1345 (%)	Sí=255 (%)	Valor de p	0=822 (%)	1=523 (%)	2=187 (%)	>3=68 (%)	Valor de p
Sexo									
	Mujer	653 (48,6)	154 (60,4)	<0,001	382 (46,5)	271 (51,8)	109 (58,3)	45 (66,2)	<0,001
	Varón	692 (51,4)	101 (39,6)		440 (53,5)	252 (48,2)	78 (41,7)	23 (33,8)	
Edad									
	30-39 años	416 (30,9)	21 (8,2)	<0,001	315 (38,3)	101 (19,4)	17 (9,1)	4 (5,9)	<0,001
	40-49 años	434 (32,3)	45 (17,7)		253 (30,8)	181 (34,6)	37 (19,8)	8 (11,7)	
	50-59 años	308 (22,9)	99 (38,8)		165 (20,1)	143 (27,3)	70 (37,4)	29 (42,7)	
	60+ años	187 (13,9)	90 (35,3)		89 (10,8)	98 (18,7)	63 (33,7)	27 (39,7)	
Nivel de educación									
	Primaria	394 (29,3)	123 (48,2)	<0,001	210 (25,6)	184 (35,2)	79 (42,3)	44 (64,7)	<0,001
	Secundaria	642 (47,7)	101 (39,6)		414 (50,4)	228 (43,6)	83 (44,4)	18 (26,5)	
	Superior	309 (23,0)	31 (12,2)		198 (24,0)	111 (21,2)	25 (13,4)	6 (8,8)	
Nivel socioeconómico									
	Bajo	446 (33,2)	92 (36,1)	0,650	267 (32,5)	179 (34,2)	65 (34,8)	27 (39,7)	0,220
	Medio	461 (34,3)	85 (33,3)		303 (36,8)	158 (30,2)	63 (33,7)	22 (32,4)	
	Alto	438 (32,5)	78 (30,6)		252 (30,7)	186 (35,6)	59 (31,5)	19 (27,9)	
Actualmente empleado									
	Sí	937 (69,7)	149 (58,4)	<0,001	577 (70,2)	360 (68,8)	121 (64.7)	28 (41,2)	<0,001
Seguro de salud									
	Sí	1224 (91,0)	238 (93,3)	0,220	746 (90,7)	478 (91,4)	170 (90,9)	68 (100,0)	0,080
Tabaquismo diario									
	Sí	81 (6,0)	11 (4,3)	0,280	54 (6,6)	27 (5,2)	8 (4,3)	3 (4,4)	0,510
Abuso de alcohol									
	Sí	111 (8,3)	8 (3,1)	0,004	78 (9,5)	33 (6,3)	6 (3,2)	2 (2,9)	0,005
Actividad física									
	Moderada/alta	860 (63,9)	138 (54,1)	0,003	534 (65,0)	326 (62,3)	107 (57,2)	31 (45,6)	0,006
	Baja	485 (36,1)	117 (45,9)		288 (35,0)	197 (37,7)	80 (42,8)	37 (54,4)	

### Características de la población según indicadores de salud mental

La prevalencia de síntomas depresivos fue de 23,3% (IC95%: 21,1% - 25,3%) y fue más frecuente en mujeres (p <0,001), en aquellos con mayor número de condiciones crónicas (p<0,001) y en los que tenían multimorbilidad (p<0,001). Por otro lado, la prevalencia de síntomas de ansiedad fue de 42,0% (IC95%: 39,5% - 44,4%) y estuvo asociado al sexo femenino (p<0,001), a la ausencia de tabaquismo (p=0,011), a no presentar abuso de alcohol (p=0,004), a un mayor número de condiciones crónicas (p<0,001) y a la presencia de multimorbilidad (p<0,001). Por último, el estrés percibido fue más frecuente en mujeres (p<0,001), en aquellos que no fumaban diariamente (p=0,003), en personas que tenían un mayor número de condiciones crónicas (p<0,001) y en los que presentaban multimorbilidad (p<0,001). Ver detalles en la Tabla 2.

**Tabla 2:** Características de la población de estudio según indicadores de salud mental (síntomas depresivos, de ansiedad y estrés).

			Síntomas depresivos		Síntomas de ansiedad		Estrés percibido	
		n	Sí=327 (%)	Valor de p	Sí=672 (%)	Valor de p	Alto=503 (%)	Valor de p
Sexo								
	Mujer	807	276 (34,2)	<0,001	452 (56,0)	<0,001	339 (42,0)	<0,001
	Varón	793	96 (12,1)		220 (27,7)		164 (20,6)	
Edad								
	30-39 años	437	95 (21,7)	0,160	173 (39,5)	0,030	130 (29,7)	0,470
	40-49 años	479	108 (22,5)		205 (42,8)		162 (33,8)	
	50-59 años	407	111 (27,2)		192 (47,1)		130 (31,9)	
	60+ años	277	58 (20,9)		102 (36,8)		81 (29,2)	
Nivel de educación								
	Primaria	517	136 (26,3)	0,140	223 (43,1)	0,590	182 (35,2)	0,002
	Secundaria	743	162 (21,8)		302 (40,7)		239 (32,2)	
	Superior	340	74 (21,8)		147 (43,2)		82 (24,1)	
Nivel socioeconómico								
	Bajo	538	132 (24,5)	0,490	232 (43,1)	0,680	190 (35,3)	0,050
	Medio	546	129 (23,6)		231 (42,3)		164(30,0)	
	Alto	516	111 (21,5)		209 (40,5)		149 (28,9)	
Actualmente empleado								
	No	514	179 (34,8)	<0,001	275 (53,5)	<0,001	216 (42,0)	<0,001
	Sí	1086	193 (17,8)		397 (36,6)		287 (26,4)	
Seguro de salud								
	No	138	27 (19,6)	0,280	49 (35,5)	0,110	30 (21,7)	0,010
	Sí	1462	345 (23,6)		623 (42,6)		473 (32,4)	
Tabaquismo diario								
	No	1508	357 (23,7)	0,10	645 (42,8)	0,010	487 (32,3)	0,003
	Sí	92	15 (16,3)		27 (29,4)		16 (17,4)	
Abuso de alcohol								
	No	1481	351 (23,7)	0,130	637 (43,0)	0,004	475 (32,1)	0,050
	Sí	118	21 (17,7)		35 (29,4)		28 (23,5)	
Actividad física								
	Moderada/alta	998	199 (19,9)	<0,001	389 (39,0)	0,002	273 (27,4)	<0,001
	Baja	602	173 (28,7)		283 (47,0)		230 (38,2)	
Número de condiciones crónicas								
	0	822	166 (20,2)	<0,001	290 (35,3)	<0,001	217 (26,4)	<0,001
	1	523	112 (21,4)		231 (44,2)		167 (31,9)	
	2	187	59 (31,6)		102 (54,6)		76 (40,6)	
	3+	68	35 (51,5)		49 (72,1)		43 (63,2)	
Multimorbilidad								
	No	1345	278 (20,7)	<0,001	521 (38,7)	<0,001	384 (28,6)	<0,001
	Sí	255	94 (36,9)		151 (59,2)		119 (46,7)	

### Asociación entre multimorbilidad y salud mental

En modelo multivariable, la presencia de multimorbilidad se asoció a una mayor prevalencia de síntomas depresivos (61%; IC95%: 32% - 98%) y a una mayor prevalencia de síntomas de ansiedad (46%; IC95%: 28% - 66%). Mientras que la presencia de multimorbilidad estuvo asociado a niveles altos de estrés (22%; IC95%: 14% - 33%), no lo estuvo para niveles moderados (6%; IC95%: 0% - 12%). De otro lado, un mayor número de condiciones crónicas se asoció a una mayor prevalencia de síntomas depresivos, síntomas de ansiedad y estrés percibido (Tabla 3).

**Tabla 3:** Asociación entre multimorbilidad, número de condiciones crónicas y salud mental: Modelos crudos y ajustados.

		Multimorbilidad	Número de condiciones crónicas		
		Sí vs. No (IC 95%)	1 vs. 0 (IC 95%)	2 vs. 0 (IC 95%)	>3 vs. 0 (IC 95%)
Síntomas depresivos					
	Modelo crudo	1,78 (1,47 - 2,16)	1,06 (0,86 - 1,31)	1,56 (1,22 - 2,01)	2,55 (1,95 - 3,33)
	Modelo ajustado*	1,61 (1,32 - 1,98)	1,03 (0,83 - 1,27)	1,44 (1,11 - 1,87)	2,14 (1,60 - 2,89)
Síntomas de ansiedad					
	Modelo crudo	1,53 (1,35 - 1,73)	1,25 (1,10 - 1,43)	1,54 (1,31 - 1,81)	2,04 (1,71 - 2,43)
	Modelo ajustado*	1,46 (1,28 - 1,66)	1,23 (1,08 - 1,40)	1,50 (1,27 - 1,77)	1,93 (1,61 - 2,32)
Estrés percibido (moderado vs. bajo)					
	Modelo crudo	1,07 (1,01 - 1,12)	0,99 (0,96 - 1,04)	1,03 (0,96 - 1,10)	1,23 (1,13 - 1,33)
	Modelo ajustado*	1,05 (0,99 - 1,12)	0,99 (0,95 - 1,04)	1,02 (0,95 - 1,09)	1,20 (1,11 - 1,30)
Estrés percibido (alto vs. bajo)					
	Modelo crudo	1,26 (1,17 - 1,34)	1,07 (0,99 - 1,15)	1,20 (1,09 - 1,31)	1,54 (1,44 - 1,66)
	Modelo ajustado*	1,22 (1,14 - 1,30)	1,05 (0,98 - 1,12)	1,16 (1,07 - 1,27)	1,49 (1,37 - 1,63)

Los modelos evalúan cada resultado (en la columna de la derecha) según cada exposición por separado (en las columnas).

* Modelo ajustado por sexo, edad, nivel de educación, nivel socioeconómico, actualmente empleado, seguro de salud, tabaquismo diario, abuso de alcohol y actividad física diaria

## DISCUSIÓN

Nuestros hallazgos muestran que tanto la multimorbilidad como un mayor número de condiciones crónicas estuvieron asociadas a una mayor prevalencia de síntomas depresivos, ansiedad y estrés percibido. De otro lado, el 15% de la población estudiada presentaba multimorbilidad, mientras que una gran proporción de la población presentaron síntomas de depresión, ansiedad y estrés percibido.

Diversos estudios muestran que la multimorbilidad se asocia a diversos problemas en la salud mental. Por ejemplo, de acuerdo a los resultados de una revisión sistemática relativamente reciente ^26^, tanto la ansiedad como la depresión fueron más frecuentes en personas con multimorbilidad. En cuanto a ansiedad, dos estudios previos corroboran nuestros hallazgos. Así, un estudio realizado en EE.UU., incluyendo 4219 adultos mayores, reportó que un mayor número de afecciones crónicas se asocian a un mayor riesgo de ansiedad ^27^. En forma similar, otro estudio que reclutó 1315 participantes con obesidad en Canadá reportó que el aumento en el número de condiciones crónicas se asoció a la presencia de síntomas de salud mental ^11^.

Respecto a depresión, un estudio demostró que el riesgo de trastorno depresivo fue dos veces mayor para las personas con multimorbilidad en comparación con aquellas sin multimorbilidad y tres veces mayor para las personas con multimorbilidad en comparación con aquellas sin ninguna condición física crónica ^10^. Dicho hallazgo es corroborado en Sudáfrica con una muestra de 2549 participantes determinó que hay una mayor prevalencia de síntomas depresivos en los que presentaban multimorbilidad ^29^.

Finalmente, en relación con el estrés percibido, de igual manera dos estudios están en línea con nuestros hallazgos. En un primer estudio de 34,129 participantes en seis países, concluyó que la multimorbilidad se asocia a mayores niveles de estrés en adultos mayores de ingresos bajos y medios ^30^. En el otro estudio donde participaron 229,293 adultos que viven en comunidades en 44 países de ingresos bajos y medios se determinó que la multimorbilidad se asoció a una mayor prevalencia de estrés percibido ^31^.

Se considera que la multimorbilidad es un reto en el campo de la salud pública, y que la afectación de la salud mental debido al incremento en el número de condiciones crónicas que está sucediendo puede tener un impacto relevante. Esto puede ser importante en países con limitados recursos como el Perú debido a la falta de un abordaje integral de las patologías que presentan los pacientes. En este caso, aunque el manejo debería ser transversal, los especialistas usualmente evalúan solo las condiciones que le son competentes sin necesidad de verificar su impacto en otros aspectos de la vida del paciente. En ese contexto, un individuo debe ser considerado como ser biopsicosocial y por lo tanto manejar apropiadamente tanto la salud física como la mental.

Nuestros resultados, al igual que otros estudios, sugieren que se debe implementar un sistema apropiado para un abordaje multidisciplinario para los pacientes con multimorbilidad. Creemos que conocer la asociación entre la presencia de múltiples condiciones crónicas y la salud mental es esencial para proporcionar una atención de salud más completa, mejorar los resultados del tratamiento y la calidad de vida de los pacientes, así como para informar las políticas y las estrategias de salud de manera efectiva.

La pandemia del COVID-19 expuso todas las deficiencias de nuestro sistema de salud que afectan el diseño de estrategias de atención, prevención y, evaluación de las políticas de salud e investigación, y estudios epidemiológicos relacionados con la multimorbilidad ^32^. Además, la pandemia puso de manifiesto la necesidad de invertir recursos en salud mental en el Perú ^33^, por lo tanto, resulta fundamental que tanto las autoridades como la sociedad en su totalidad reconozcan la necesidad de abordar los desafíos relacionados con la salud mental de manera completa y eficaz.

Una fortaleza importante del presente estudio radica en su representatividad poblacional y en la evaluación objetiva de varias de las condiciones crónicas incluidas, especialmente en aquellas más prevalentes. Sin embargo, existen algunas limitaciones que merecen discusión. En primer lugar, al ser un estudio transversal solo se puede evaluar asociación y no causalidad. Adicionalmente, la causalidad reversa podría estar presente, es decir que la presencia de síntomas de salud mental podría incrementar la probabilidad de tener multimorbilidad. En segundo lugar, la lista de enfermedades utilizada para la definición de multimorbilidad puede llegar a ser muy diferente en otras regiones, lo que afectaría los resultados, sin embargo, se agrupó usando la definición de multimorbilidad que es ampliamente reconocida, además de ser evaluada de acuerdo al número de condiciones crónicas. Más aún, algunas condiciones crónicas fueron autorreportadas lo que podría tener un impacto en las estimaciones. Sin embargo, muchas de ellas fueron las menos comunes a nivel poblacional (arritmia, insuficiencia cardiaca, cáncer e infarto agudo de miocardio). Finalmente, existen más enfermedades que abarcan la salud mental y que pueden estar relacionadas con la multimorbilidad como psicosis, trastorno de estrés postraumático (TEPT) y trastornos por abuso de sustancias, que no han sido evaluadas en el presente trabajo.

En conclusión, nuestros resultados muestran que la presencia de multimorbilidad estuvo asociada a una mayor prevalencia de síntomas depresivos, de ansiedad y de estrés percibido. Por otro lado, un mayor número de condiciones crónicas también se asocia a una mayor presencia de estos síntomas. Estos resultados sugieren la necesidad de un manejo adecuado de la salud mental en pacientes con multimorbilidad.
